# Stellae-123 gene expression signature improved risk stratification in Taiwanese acute myeloid leukemia patients

**DOI:** 10.1038/s41598-024-61022-5

**Published:** 2024-05-14

**Authors:** Yu-Hung Wang, Adrián Mosquera Orgueira, Chien-Chin Lin, Chi-Yuan Yao, Min-Yen Lo, Cheng-Hong Tsai, Adolfo de la Fuente Burguera, Hsin-An Hou, Wen-Chien Chou, Hwei-Fang Tien

**Affiliations:** 1 Division of Hematology, National Taiwan University Hospital, Taipei, Taiwan; 2 Department of Hematology, University Hospital of Santiago de Compostela, Santiago de Compostela, Spain; 3 Group of Computational Hematology and Genomics (GrHeCo-Xen), Instituto de Investigación Sanitaria de Santiago de Compostela (IDIS), Santiago de Compostela, Spain; 4 Department of Laboratory Medicine, National Taiwan University Hospital, No. 7, Chung-Shan S. Rd., Taipei City, 10002 Taiwan; 5 Graduate Institute of Clinical Medicine, College of Medicine, National Taiwan University, Taipei, Taiwan; 6 Division of Hematology, Department of Internal Medicine, National Taiwan University Hospital Yunlin Branch, Yunlin, Taiwan; 7 Department of Medical Education and Research, National Taiwan University Hospital Yunlin Branch, Yunlin, Taiwan; 8 Department of Hematology, Hospital MD Anderson Cancer Center Madrid, Madrid, Spain; 9 Department of Internal Medicine, Far-Eastern Memorial Hospital, No. 7, Chung-Shan S. Rd., Taipei City, 10002 Taiwan

**Keywords:** Acute myeloid leukaemia, Bioinformatics, Cancer, Data mining

## Abstract

The European Leukemia Net recommendations provide valuable guidance in treatment decisions of patients with acute myeloid leukemia (AML). However, the genetic complexity and heterogeneity of AML are not fully covered, notwithstanding that gene expression analysis is crucial in the risk stratification of AML. The Stellae-123 score, an AI-based model that captures gene expression patterns, has demonstrated robust survival predictions in AML patients across four western-population cohorts. This study aims to evaluate the applicability of Stellae-123 in a Taiwanese cohort. The Stellae-123 model was applied to 304 de novo AML patients diagnosed and treated at the National Taiwan University Hospital. We find that the pretrained (BeatAML-based) model achieved c-indexes of 0.631 and 0.632 for the prediction of overall survival (OS) and relapse-free survival (RFS), respectively. Model retraining within our cohort further improve the cross-validated c-indexes to 0.667 and 0.667 for OS and RFS prediction, respectively. Multivariable analysis identify both pretrained and retrained models as independent prognostic biomarkers. We further show that incorporating age, Stellae-123, and ELN classification remarkably improves risk stratification, revealing c-indices of 0.73 and 0.728 for OS and RFS, respectively. In summary, the Stellae-123 gene expression signature is a valuable prognostic tool for AML patients and model retraining can improve the accuracy and applicability of the model in different populations.

## Introduction

The European Leukemia Net (ELN)-2022 risk stratification system for acute myeloid leukemia (AML) provides valuable guidance in the management of adult AML patients^1^. Nonetheless, by stratifying patients into three risk categories, the genetic complexity and heterogeneity of the disease are not fully covered by the ELN system^2^. Rare or newly discovered mutations that could impact prognosis are not taken into account, and there is an unmet need for a dynamic assessment of changing disease status and treatment response within the system^3^. Additionally, the system's predictive accuracy may vary for individual patients due to its limited integration of clinical factors, such as those reported in a recent research^4^, underscoring the necessity for further research and personalized approaches to optimize patient care in AML^5,6^. Addressing these gaps requires ongoing research, real-world validation, and the integration of a more comprehensive understanding of AML.

Gene expression data provides an invaluable resource for risk stratifying AML patients. Transcriptomic changes are associated with mutations, cytogenetic abnormalities, and signaling pathway alterations^7^. Gene expression analysis has unveiled distinct gene expression patterns and their associations with prognostic factors, including biological age and molecular subtypes, allowing for improved risk stratification in AML patients^8–12^.

Stellae-123, a machine learning model based on gene expression patterns, initially demonstrated precise and accurate personalized survival predictions in adult AML patients^13^. This model originally included 123 variables, encompassing the expression of 121 genes. Afterwards, a reduced version of Stellae-123 based on 69 genes consistently demonstrated robust predictive power across various cohorts^14^, achieving c-indexes of 0.64, 0.65, and 0.60 for overall survival (OS) prediction in the BeatAML, AMLCG-2008, and TARGET AML cohorts, respectively^15–17^. Stellae-123 effectively stratified patients with high-risk mutations, such as *ASXL1*, *RUNX1*, *TP53* and *U2AF1* mutations, into distinct prognostic groups. Essentially, these findings supported the utility of Stellae-123 as an additional prognostic tool in AML, complementing cytogenetic and mutational parameters by capturing transcriptomic changes arising from complex somatic events.

The primary objective of this study was to comprehensively validate and rigorously assess the prognostic value of the Stellae-123 gene expression signature in predicting OS and relapse-free survival (RFS) outcomes among a cohort of patients with de novo AML diagnosed and treated in a Medical Center in Taiwan. Furthermore, the study sought to investigate the potential advantages of recalibrating and retraining the predictive model utilizing the specific Taiwanese dataset, thereby accounting for inherent heterogeneity and aiming to enhance the accuracy and reliability of prognostic predictions in this distinct population.

## Methods

### Patients and treatment modalities

A total of 304 de novo AML patients diagnosed and treated at the National Taiwan University Hospital (NTUH) who had ever received standard induction with 7 + 3 chemotherapy (or 5 + 2 for elder fit patients)^18,19^ and had adequate bone marrow samples for DNA and RNA sequencing at diagnosis were included. AML was diagnosed according to the 2022 World Health Organization (WHO) classification^20^ and The International Consensus Classification of Myeloid Neoplasms and Acute Leukemias^21^. Patients with acute promyelocytic leukemia, AML with other precedent myeloid neoplasms, and therapy-related myeloid neoplasms were not included. In NTUH, patients who achieve first complete remission (CR) usually undergo consolidation therapy with two to four courses of high-dose cytarabine (2000 mg/m2 q12h, total eight doses) with or without an anthracycline (Idarubicin or Mitoxantrone)^22^, or bridged to allogeneic hematopoietic stem cell transplantation (allo-HSCT) if indicated and eligible. Clinical data including age at diagnosis, sex, hemogram, biochemistry, treatment regimen and response, allo-HSCT status, and survival were collected. The NTUH Research Ethics Committee approved the study (#201709072RINC). Informed consents were obtained in accordance with the Helsinki Declaration.

### Cytogenetic study and molecular mutation analysis by targeted next-generation sequencing (NGS)

Cytogenetic analysis was performed using bone marrow cells harvested within 3 days of unstimulated culture and metaphase chromosomes were banded via the trypsin-Giemsa banding technique. Results were categorized using the International System for Human Cytogenetic Nomenclature. Detailed methods have been previously described^23^. Gene mutations were examined via targeted NGS, using the TruSight myeloid sequencing panel (Illumina, San Diego, CA, USA), which included 15 full exon genes and 39 oncogenic hotspot genes. HiSeq platform (Illumina, San Diego, CA, USA) was used for sequencing with a median reading depth of 12000x. Owing to suboptimal sequencing sensitivity, *FLT3-*ITD and *CEBPA* mutations were confirmed by polymerase chain reaction followed by Sanger sequencing^24,25^.

### Library preparation and RNA sequencing

In total, BM samples of 304 patients were submitted for RNA sequencing. The TruSeq Stranded mRNA Library Prep Kit (Illumina, San Diego, CA, USA) was used for library preparation as previously described^26^. For more detailed information, please see the Supplemental Method.

### Validation of the pretrained Steallae-123 risk score in Taiwanese patients

Gene expression values were normalized to fragments per kilobase of transcript per million mapped reads (FPKM) values. Then, random survival forests were built to predict survival in the BeatAML cohort, as described previously^14,27^. Random forests are a machine learning algorithm that builds an ensemble of decision trees by randomly sampling the data and features, and combining the results of the individual trees to make predictions. The main objective of random forests is to increase the accuracy and robustness of predictions by reducing overfitting and variance. Random survival forests extend the random forests algorithm to handle survival data, where the outcome of interest is the time until an event of interest occurs, such as death or failure. In a random survival forest, each decision tree represents a survival model, where the outcome is the time to event and the predictors are the input features. The final prediction is then made by aggregating the results of all decision trees in the random survival forest. The algorithm was tuned with 1,000 trees and standard predefined parameters. The resulting model was used to obtain cumulative hazard risk predictions from the Taiwanese cohort based on the previous training in the BeatAML cohort. The discriminative capacity of this model was evaluated using Harrel’s c-indexes.

### Evaluation of model retraining within the Taiwanese cohort

We then explored if the same transcripts used to construct Stellae-123 could further improve prognostication in the Taiwanese cohort using model retraining. Machine learning model retraining refers to the process of updating and improving an existing machine learning model by incorporating new data. When a model is initially trained, it learns patterns and relationships in the training data to make predictions or classifications. However, as new data becomes available over time, the model may become less accurate or fail to adapt to changing patterns in the data. In the particular case, differences in population structure, diagnostic procedures and treatment protocols might have an impact on patient outcomes. Retraining of the model can adapt the performance of the model to the particular characteristics of a different health system. Random survival forest retraining was performed with default hyperparameters and 1,000 trees.

### Statistical analysis

The Fisher's exact test or the Chi-square test were used to compare categorical or nominal variables. To compare continuous variables, Mann–Whitney or Kruskal–Wallis tests were used. Response criteria and definition of clinical outcome, including complete remission (CR), relapse or refractory disease, OS, and RFS follow the ELN-2022 recommendation^1^. The Kaplan–Meier method was used to calculate the chance of survival, and the log-rank test was used to assess differences. Landmark analysis was conducted to exclude the impact of early mortality. For univariate and multivariable analysis, the Cox proportional hazard model was employed.

## Results

### Patient characteristics

Baseline demographics and mutation profiles of patients are summarized in Table 1 and Table S1–S3. The median age of the 304 AML patients was 46 years. Overall, 137 (45%), 82 (27%), and 85 (28%) patients were classified into ELN-2022 favorable, intermediate, and adverse risk groups, respectively. A total of 208 (68.4%) patients achieved CR after induction chemotherapy while 110 (36.2%) patients received allo-HSCT. With a median follow up of 23 months, 136 (44.7%) patients experienced relapse of the disease and 196 (64.4%) patients succumbed to the disease.

**Table 1:** Comparison of clinical and laboratory features between 304 AML patients stratified by Stellae-123 model derived from the BeatAML cohort.

Variable [median (range) or n (%)]	Total	Stellae-123 Risk Group			*P* value
		Favorable (n = 101)	Intermediate (n = 101)	Adverse (n = 102)	
Age (years)	46 (18–86)	44 (18–78)	46 (18–86)	47 (18–84)	NS
Sex, Male	164 (53.9)	50 (49.5)	55 (54.5)	59 (57.8)	NS
Laboratory data					
White Blood Cell (× 10^9^/L)	34.2 (1.1–406)	38.5 (1.5–324)	35.8 (1.1–406)	30.1 (1.2–341)	NS
Hemoglobin (g/dL)	8.0 (2.7–13.6)	8.3 (2.7–13.6)	8.1 (4.2–13.6)	7.5 (3.7–13.2)	NS
Platelet (× 10^9^/L)	45 (3–751)	41 (5–203)	45 (3–712)	51 (3–751)	NS
Peripheral Blood Blasts (%)	54 (0–99)	50 (0–99)	49 (0–99)	64 (0–99)	NS
Lactate Dehydrogenase (U/L)*	1042 (194–8693)	997 (291–6711)	1119 (194–8280)	1010 (250–8693)	NS
FAB Category					0.039
M0	3 (1)	0	0	3 (29)	
M1	83 (27.3)	33 (32.7)	18 (17.8)	32 (31.4)	
M2	110 (36.2)	33 (32.7)	42 (41.6)	35 (34.3)	
M4	84 (27.6)	29 (28.7)	34 (33.7)	21 (20.6)	
M5	16 (5.3)	4 (4)	6 (5.9)	6 (5.9)	
M6	8 (2.6)	2 (2.0)	1 (1.0)	5 (4.9)	
ELN-2022 risk category					< 0.001
Favorable	137 (45.0)	76 (75.2)	44 (43.6)	17 (16.7)	
Intermediate	82 (27.0)	20 (19.8)	38 (37.6)	24 (23.5)	
Adverse	85 (28.0)	5 (5.0)	19 (18.8)	61 (59.8)	
Response of induction					< 0.001
Complete remission	208 (68.4)	85 (84.2)	75 (74.3)	48 (47.1)	
No response	96 (31.6)	16 (15.8)	26 (25.7)	54 (52.9)	
Relapse	136 (44.7)	45 (44.6)	47 (46.5)	44 (43.1)	NS
Death during/after induction	25 (8.2)	5 (5.0)	9 (8.9)	11 (10.8)	NS
Allogeneic hematopoietic stem cell transplantation	110 (36.2)	42 (41.6)	31 (30.7)	37 (36.2)	NS

### Application of Stellae-123 to the Taiwanese cohort

**Figure 1:**
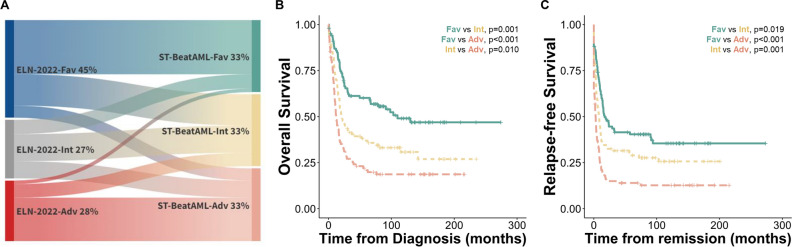
Regrouping of patients and survival outcomes according to Stellae-123 risk groups in the pretrained model (BeatAML). (**A**) Sankey diagram showing re-distribution of patients from European LeukemiaNet (ELN)-2022 risk groups into Stellae-123 risk groups (pretrained model). Kaplan–Meier survival curves of overall survival (**B**) and relapse-free survival (**C**) of Taiwanese patients according to their risk predictions by the pretrained Stellae-123 model (BeatAML). Patients were assigned to tertiles of risk for graphical representation. Fav: favorable, Int: intermediate, Adv: adverse; and ST-BeatAML, Stellae-123 pretrained model base on the BeatAML study.

The original variables of the Stellae-123 gene expression signature were FPMK counts from RNA-seq data. We identified these variables and selected them in the Taiwanese RNA-seq data set. This ended up in the construction of a 69-gene expression matrix per patient. The original model was trained in the BeatAML cohort (USA), where it achieved a c-index of 0.635. We applied the original model to the Taiwanese cohort. Patients were stratified into favorable-, intermediate-, and adverse-risk groups, each representing a tertile of the cohort. Overall, there was no difference in clinical variables including age, sex, and laboratory parameters among groups (Table 1). Intersecting with the ELN risk stratification, Stellae-123 risk groups partly aligned with ELN-2022 risk groups (Table 1 and Fig. 1A). Overall, 42.4% of the patients in the total cohort were regrouped into different risk categories from the ELN-2022 to the Stellae-123 system (favorable: 44.5%; intermediate: 53.7%; and adverse: 28.2%).

### Validation of the Stellae-123 for OS and RFS prediction

We initially assessed the effectiveness of ELN-2022 in risk stratification. The patient outcomes stratified by ELN-2022 risk categories (Fig. S1) revealed c-indices of 0.644 and 0.654 for OS and RFS, respectively. However, the observed survival disparity between the intermediate- and adverse-risk groups was only modest (log-rank *p*-values of 0.057 and 0.215 for OS and RFS, respectively, Fig. S1).

We next extracted the cumulative hazards predicted by Stellae-123 for OS from each patient. Univariate analysis demonstrated the prognostic significance of Stellae-123 score, with a hazard ratio (HR) of 1.023 (95% confidence interval [CI]: 1.015–1.031, *P* < 0.001). The c-index of this score was 0.631 for OS prediction, and the division of the cohort in 3 equal groups evidenced the divergent outcomes of each group (Fig. 1B). Similar to what was observed previously^14^, the molecular predictor was especially effective for risk-stratification after the initial months post-diagnosis, which might be explained by the fact that most early deaths are related to events not related to genomic aberrations (e.g., treatment toxicity and infections).

Since Stellae-123 is based on molecular features from the leukemic cells, we expected that it could also be a useful predictor of RFS. To test this hypothesis, we calculated the accuracy of the cumulative hazards calculated by Stellae-123 to predict RFS, yielding a c-index of 0.632 and a HR of 1.02 (95%CI: 1.012–1.028, *P* < 0.001). In this case, the division of the cohort in 3 equal groups (each representing 33% of the cohort) evidenced the divergent risk of relapse of each group of patients (Fig. 1C). While the c-indices of Stellae-123 were slightly lower than those of ELN-2022 stratification, the discriminatory ability between the Stellae-123 intermediate- and adverse-risk groups proved superior (OS, *P* = 0.01; and RFS, *P* = 0.001, respectively, Fig. 1B&C).

### Retraining of the algorithm in the Taiwanese population

**Figure 2:**
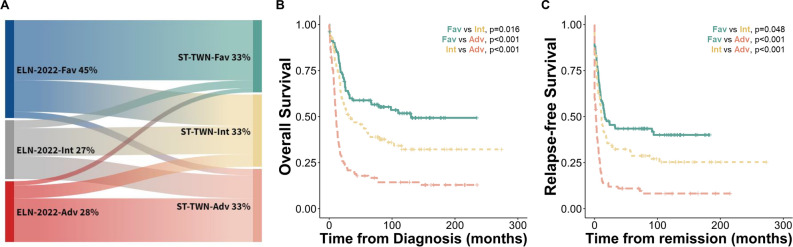
Regrouping of patients and survival outcomes according to Stellae-123 risk groups in the retrained model (Taiwan). (**A**) Sankey diagram showing re-distribution of patients from European LeukemiaNet (ELN)-2022 risk groups into Stellae-123 risk groups (retrained model). Kaplan–Meier survival curves of overall survival (**B**) and relapse-free survival (**C**) of Taiwanese patients according to their risk predictions by the retrained Stellae-123 model (Taiwan). Patients were assigned to tertiles of risk for graphical representation. Fav: favorable, Int: intermediate, Adv: adverse; and ST-TWN, Stellae-123 retrained model base on the Taiwanese transcriptomic data.

We hypothesized that there might be substantial variation between the original training cohort of the model and the Taiwanese population, including both at the molecular level (e.g., differences in population genetics) and in the healthcare system that could affect the outcomes of AML patients. In light of this, we wondered how much model retraining would improve the cross-validated results in the Taiwanese population. The distribution of patients in the retrained model risk groups is displayed in Fig. 2A. Compared to the pretrained model (Stellae-123 BeatAML), nine more patients in the retrained model (Stellae-123 Taiwan) shared the same risk category in the ELN system. In total, the risk categories of 120 (39.5%) patients were changed: favorable, 38.7%, intermediate, 52.4%, and adverse, 28.2%. Regarding prognostication, univariate analysis reaffirmed the prognostic discriminative ability of the retrained Stellae-123 model for both OS and RFS (OS: HR 1.015 [1.011–1.019], *P* < 0.001; and RFS: HR 1.014 [1.011–1.018, *P* < 0.001], respectively). The cross-validated c-indexes for OS and RFS prediction were 0.667 each (Fig. 2B&C), indicating the potential applicability of the locally retrained model across diverse regions.

**Figure 3:**
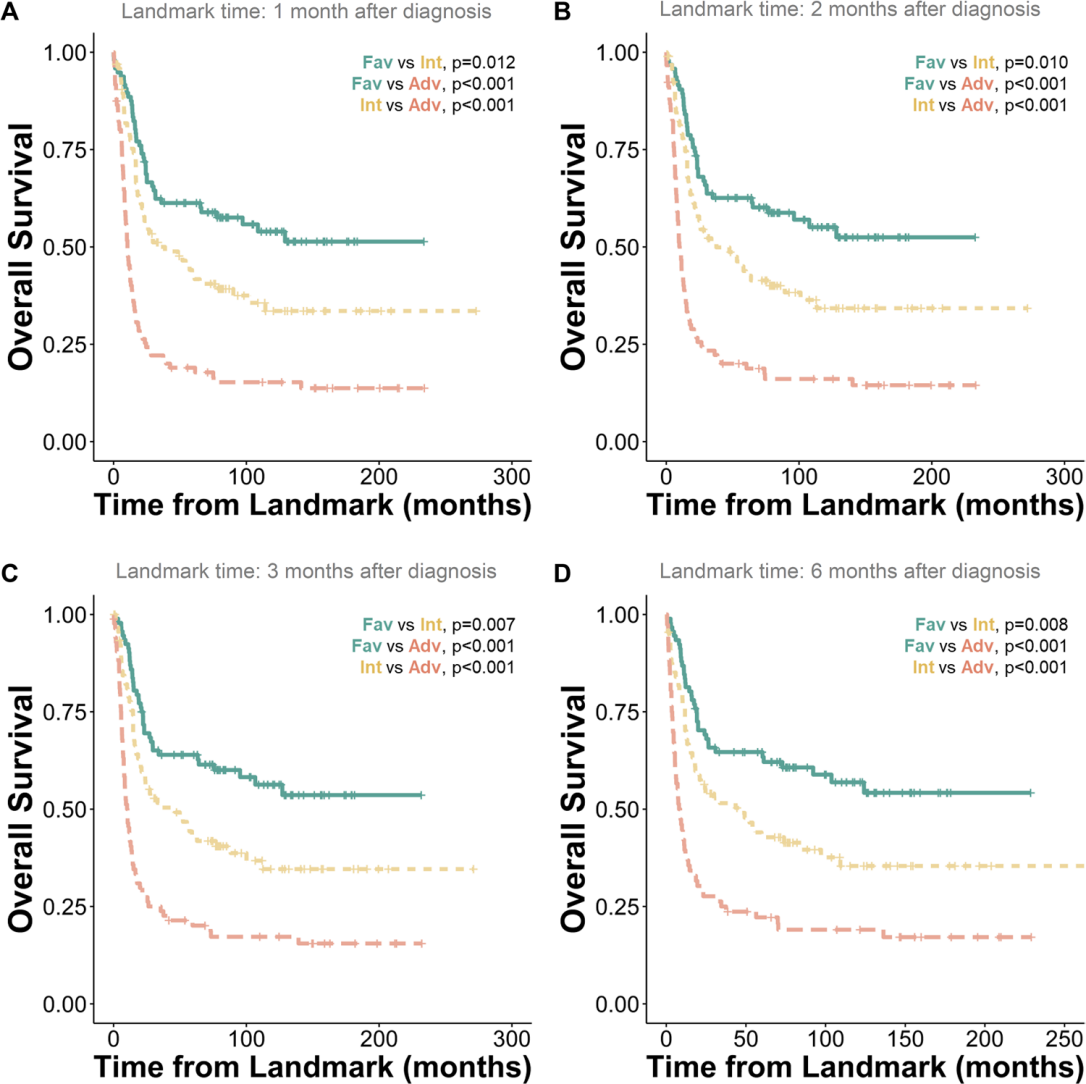
Landmark analysis demonstrating overall survival in the retrained Stellae-123 model (Taiwan). Landmark time was set at 1 (**A**), 2 (**B**), 3 (**C**), and 6 (**D**) months after diagnosis, respectively.

To exclude the impact of early mortalities, we conducted a landmark analysis, setting the time points at 1, 2, 3, and 6 months post-diagnosis, resulting in the exclusion of 15, 24, 34, and 49 patients, respectively (Table S4). This refined approach further enhanced the prognostic capabilities of the retrained model (Stellae-123 Taiwan), yielding c-indexes up to 0.681 for OS prediction (Table S4). Notably, patients' OS and RFS were more effectively stratified by tertiles (Fig. 3A-D and Fig. S2), primarily enhancing the distinction between favorable and intermediate groups, thereby affirming the robustness of the retrained model.

### Multivariable analysis

Given the partially overlapped grouping with ELN-2022 in both the pretrained and retrained models (favorable: > 55%; intermediate: > 45%; and adverse: > 70%, respectively), we wondered whether Stellae-123 models could stratify patients in an ELN-risk-independent manner. Adjusted with age and ELN-2022 in the multivariable analysis, both the pretrained and retrained Stellae-123 models, either calculated as continuous values (Table 2) or divided by 3 groups (Table S5), consistently showed discriminative power in OS and RFS prognostication.

**Table 2:** Multivariable analysis for overall survival (OS) and relapse-free survival (RFS) in the 304 AML patients, using the pretrained model (Stellae-123 BeatAML) (upper panel) and the retrained model (Stellae-123 Taiwan) (lower panel).

	RFS				OS			
	HR	95%CI		*P*	HR	95%CI		*P*
Pretrained model (Stellae-123 BeatAML)								
Age	1.025	1.017	1.034	< 0.001	1.030	1.020	1.039	< 0.001
ELN-2022								
Fav (vs Adv)	0.518	0.351	0.765	0.001	0.476	0.316	0.717	< 0.001
Int (vs Adv)	1.080	0.756	1.542	0.672	0.998	0.693	1.437	0.990
Stellae-123 (Pretrained)*	1.011	1.002	1.021	0.016	1.012	1.003	1.022	0.011

	RFS				OS			
	HR	95%CI		*P*	HR	95%CI		*P*
Retrained model (Stellae-123 Taiwan)								
Age*	1.025	1.016	1.034	< 0.001	1.029	1.019	1.038	< 0.001
ELN-2022								
Fav (vs Adv)	0.651	0.440	0.963	0.032	0.586	0.387	0.887	0.011
Int (vs Adv)	1.069	0.763	1.497	0.699	0.972	0.685	1.379	0.873
Stellae-123 (Retrained)*	1.011	1.006	1.015	< 0.001	1.010	1.006	1.015	< 0.001

Statistically significant if *P* < 0.05.

*As continuous variable.

HR, hazard ratios; CI, confidence interval.

### Incorporating age as a covariate for risk stratification

**Figure 4:**
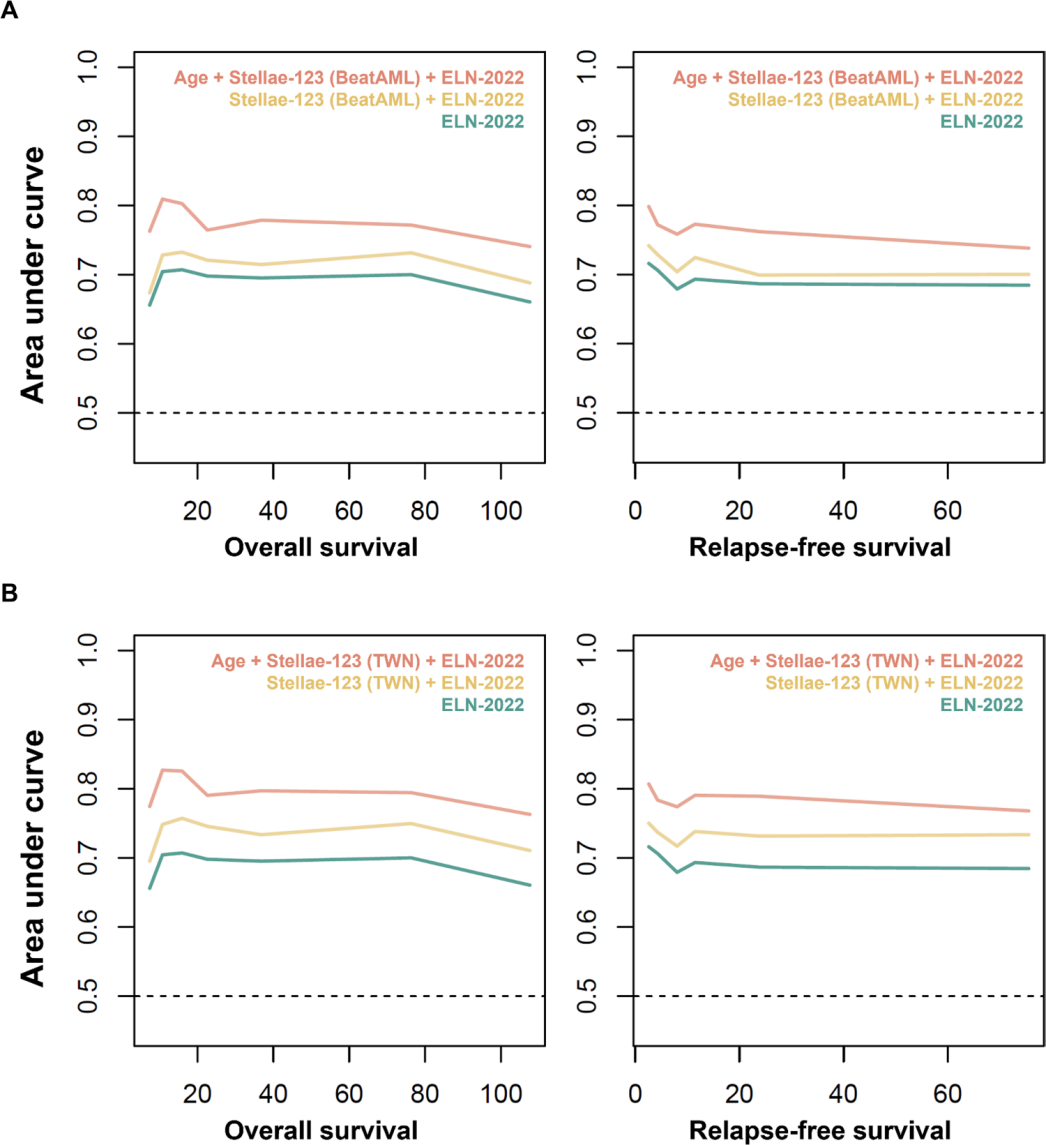
Time-dependent ROC curve analyses demonstrate that Stellae-123 and age can be complementary to current risk stratification. Both pretrained (BeatAML) (**A**) and retrained (Taiwan) (**B**) Stellae-123 models could be complementary to ELN-2022 risk stratification when incorporated. Taking age into consideration further improved the power of prognostication.

In light of recent data showing that incorporating age helped improve the performance of prognostication of ELN-2022^28^ and the fact that age was shown to be an independent risk factor for inferior outcome in our cohort, we next examined how age could complement current risk stratification. A serial of model testing revealed that, on the basis of ELN-2022, taking the transcriptomic data into consideration, particularly the locally retrained model, could robustly improve the prognostic models (Table S6) with significant declines of delta Akaike Information Criterion (AIC) values. Furthermore, in both pretrained and retrained models, incorporating age similarly further refined these models for OS and RFS prognostication. Time-dependent ROC curve analysis also indicated the potential of incorporating Stellae-123 (both pretrained and retrained) and age to complement prognostic performance of ELN-2022 (Fig. 4A&B).

**Figure 5:**
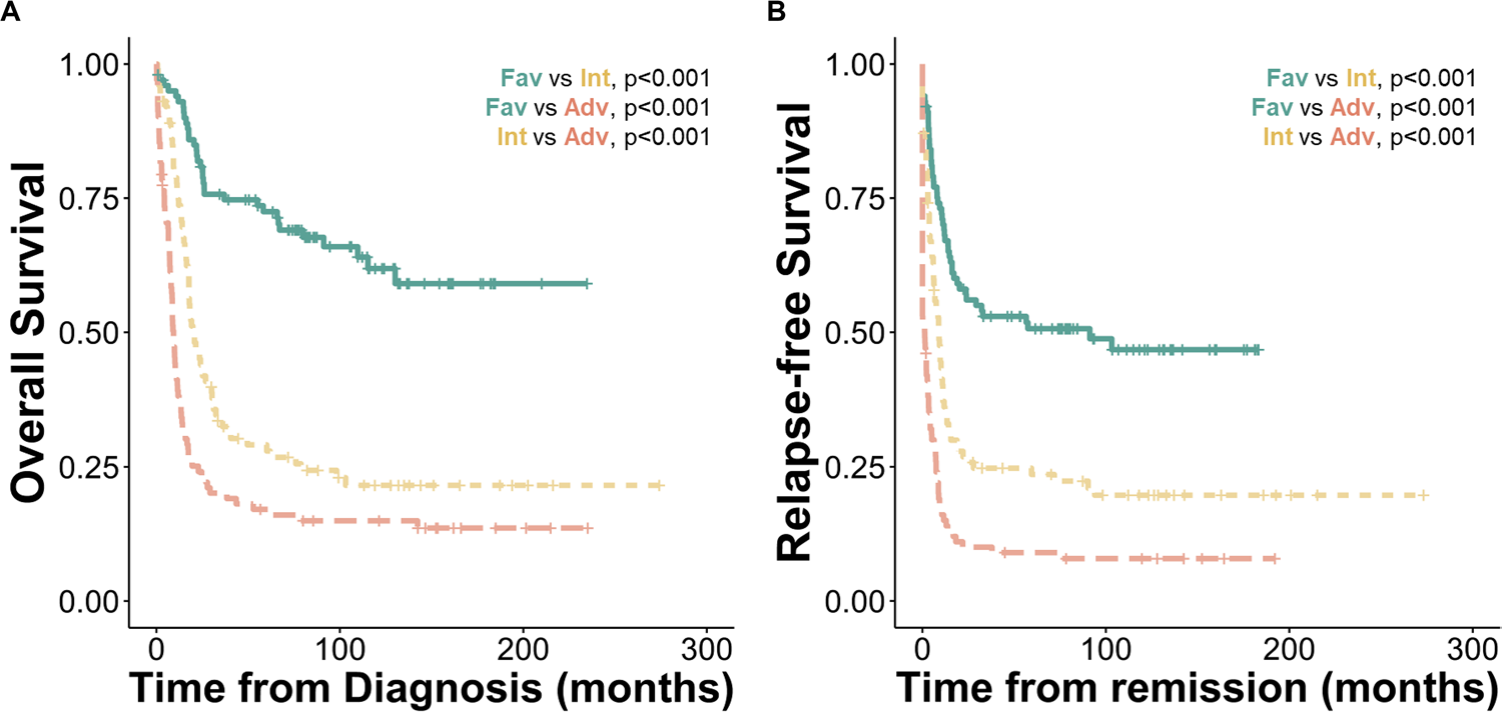
Improved prognostic systems incorporating European LeukemiaNet (ELN)-2022 risk stratification, retrained Stellae-123 model (Taiwan), and age. Kaplan–Meier survival curves of overall survival (OS) (**A**) and relapse-free survival (RFS) (**B**) of Taiwanese patients according to the risk groups incorporating ELN risk groups, retrained Stellae-123 model (Taiwan), and age.

Indeed, the c-indices for OS and RFS, assessed using the prognostic system that incorporates ELN-2022, age, and the retrained Stellae-123 model (ELN/Age/AI), were further elevated to 0.73 and 0.728, respectively. Moreover, as depicted in Fig. 5, patients' OS and RFS were robustly stratified by this ELN/Age/AI system, with log-rank *p*-values less than 0.001 in each comparison. Similarly, a landmark analysis was executed to evaluate the influence of early deaths within the ELN/Age/AI system. This analysis resulted in bolstered c-indexes of 0.734, 0.739, and 0.733 when assessing landmark times at 1, 2, and 3 months post-diagnosis, respectively (Table S7), accompanied by distinct separation of patients' OS and RFS curves (Fig. S3), suggesting its potential utility in refining prognostic assessments and guiding clinical decision-making.

**Figure 6:**
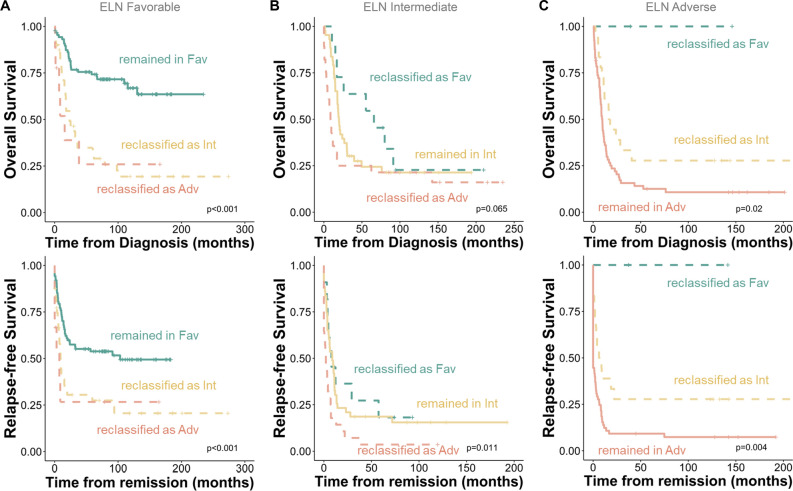
Outcome evaluation of patients who were reclassified from European LeukemiaNet (ELN)-2022 to the ELN/Age/AI (Stellae-123 retrained model) system. Kaplan–Meier survival curves depict outcomes of patients remaining in ELN favorable- (**A**), intermediate- (**B**), and adverse-risk (**C**) groups, along with those reclassified.

Subsequently, we compared the outcomes of patients who were reclassified between the ELN and the ELN/Age/AI system with those who remained in the same risk categories. As illustrated in Fig. 6A, ELN favorable-risk patients reclassified to intermediate or adverse risk groups experienced significantly shorter OS and RFS than those who remained in the favorable-risk group. For the ELN intermediate- and adverse-risk groups, patients reclassified to better-risk groups had longer survival than those remaining in the same risk group, while the opposite was true for those reclassified to poorer-risk groups (Fig. 6B&C).

To gain a deeper insight into the extent of additional information that the ELN/Age/AI system contributed to, we analyzed the mutation distribution within this system (Table S8). The enrichment of certain genes mirrors the influence of ELN classification, such as the prevalence of *CEBPA* and *KIT* mutations in the favorable-risk group and *ASXL* and *TP53* mutations in the adverse-risk group. Meanwhile, the higher frequencies of *DNMT3A* and *IDH2* mutations in the adverse-risk group might suggest biological implications not fully captured by the current system, although more evidence is required to justify their assignment to an ELN prognostic group^1^.

## Discussion

In this study, the prognostic implication of Stellae-123 gene expression signature was validated in an AML cohort from Taiwan. The original model was trained in an US cohort (BeatAML) and achieved a c-index of 0.635 for OS prediction. When applied to the Taiwanese cohort, the model exhibited a c-index of 0.631 and effectively stratified patients into three distinct risk groups. The Stellae-123 signature showed better performance in risk stratification after the initial months post-diagnosis, which is likely due to the fact that early deaths were primarily related to other factors such as toxicity and infections. Additionally, the study investigated the predictive capability of Stellae-123 for RFS and obtained a c-index of 0.632, further demonstrating its value in assessing biological risk and relapse probability. To account for potential heterogeneity between training cohorts and the Taiwanese population, the model was retrained specifically for the Taiwanese cohort. The retrained model achieved improved cross-validated c-indexes of 0.667 for OS prediction and 0.667 for RFS prediction. These findings highlight the robustness and potential clinical applicability of the Stellae-123 signature in various AML populations. Significantly, our study also demonstrates that the inclusion of age can enhance risk stratification in AML patients.

In recent years, there has been a growing recognition of the need to incorporate multiple layers of biological complexity in AML prognostication. Traditionally, prognostication in AML has relied on clinical parameters, cytogenetic abnormalities, and specific genetic mutations. While these markers provide valuable insights into patient outcomes, the complexity of AML biology cannot be fully captured by these factors^2,3^. To pursue more accurate prognostication and personalized treatment strategies, the integration of additional layers of biological information is being explored, including transcriptomic data. Transcriptomic profiling, which involves the expression levels of specific genes or gene sets was reported to provide a more comprehensive view of the functional state of leukemia cells^7,29–31^. However, the successful implementation of these approaches requires robust and standardized methodologies, large-scale data integration, and validation across diverse patient cohorts. Leveraging the AI model enables us to achieve this in a more comprehensive and unbiased way.

Combining RNA profiling with DNA mutation detection in the risk stratification of AML patients holds great promise in enhancing our understanding of the disease at a molecular level. This integration allows for a comprehensive depiction of AML's molecular landscape. DNA mutations serve as crucial biomarkers for identifying specific genetic alterations that drive the initiation and progression of AML, providing insights into the underlying genomic aberrations^15^. On the other hand, RNA profiling enables the examination of gene expression patterns, reflecting the dynamic functional state of cells and shedding light on dysregulated biological pathways. The combination of DNA mutation detection and RNA profiling offers a synergistic approach that yields a deeper understanding of the heterogeneity and underlying biology of AML^32^.

Moreover, with the routine inclusion of RNA fusion genes in diagnostic NGS solutions for AML, expanding RNA profiling is feasible^33^. Leveraging existing infrastructure and expertise in RNA fusion gene detection allows for additional layers of information regarding gene expression signatures. This expanded RNA analysis can further refine risk stratification models and optimize treatment selection. Ultimately, integrating RNA profiling and DNA mutation detection in risk stratification will empower clinicians to make better-informed decisions. By comprehensively characterizing the genomic and transcriptomic features of AML, clinicians will tailor treatment approaches to individual patients, improving therapeutic outcomes and advancing precision medicine in AML^34^.

One vital consideration in implementing machine learning models in medicine is the potential bias introduced when training on specific patient populations. These models are often trained on data from particular geographic or socioeconomic origins, limiting their generalizability and performance in diverse cohorts^35,36^. This limitation stems from variations in population characteristics, disease prevalence, genetic diversity, and healthcare practices across regions or socioeconomic backgrounds^37,38^.

To ensure the optimal performance and clinical applicability of machine learning models in diverse healthcare settings, it is critical to retrain these models with data from varied cohorts^39^. Our study exemplifies this through the retraining of the Stellae-123 model using data from a Taiwanese population. This retraining tailored the model to reflect the unique genetic and clinical nuances of this cohort, enhancing the precision and accuracy of its prognostic predictions. Such an approach is crucial for population-specific patterns, risk factors, and biomarkers pertinent to the population in question, which might not be evident in the original training dataset. Moreover, this process contributes to the equitable application of healthcare technologies, ensuring that the benefits of such models are accessible across diverse patient demographics, thereby mitigating the risk of bias towards any particular group.

Nevertheless, the process of retraining models with data from different cohorts necessitates meticulous attention to ensure its success. This involves a careful assessment of the differences in data collection methods, the quality of the data, and the potential confounding factors that might exist among various cohorts. In our study, the retraining of Stellae-123 on the Taiwanese cohort was undertaken with these considerations in mind, ensuring that the adapted model not only retained its robustness but also gained enhanced relevance and applicability to the specific population. This process underscores the importance of adapting AI models to local contexts, thereby maximizing their utility and reliability in a global healthcare landscape.

While there are some potential advantages to incorporating AI models, certain areas still require improvement. For instance, although transcriptome data offers a wealth of information, the timely and comprehensive inclusion of newly discovered transcripts may be a challenge. Additionally, the broader applicability and dynamic assessment of AI models that utilize transcriptomic profiles await further exploration and implementation.

Some limitations of the current study include the relatively limited sample size, particularly within the field of AML research, and our inability to include patients with a history of preceding myeloid neoplasia, primarily due to resource constraints such as limited biobanking and RNA sequencing availability. Nevertheless, it is noteworthy that one-fifth of the patients in our cohort exhibited myelodysplasia-related gene mutations or cytogenetic abnormalities (Table S1), thereby partially representing the currently defined "myelodysplasia-related" AML population. Additionally, our cohort is characterized by a higher proportion of ELN favorable-risk patients, resembling a miniature version of our previously published cohort^40^, yet distinct from others^41,42^, which could partially lead to a relatively lower HSCT rate. Thus, while the incorporation of age and the Stellae-123 models can complement ELN-risk assessment in our cohort, the broader applicability of our findings necessitates further validation. Moreover, although it is intriguing and crucial to assess the influence of novel agents, especially given the recent approvals in AML treatment, the timeframe of our study cohort spans from 1995 to 2011. Accordingly, our focus was directed toward this homogeneous population, all of whom received traditional induction chemotherapy. Lastly, it is important to acknowledge the retrospective nature of our study, which may introduce additional confounding factors, for instance, the heterogeneity in reinduction protocols and the diversity of responses among patients who fail to achieve complete remission after standard induction treatment.

Despite the aforementioned limitations, our study confirms the predictive value of Stellae-123 for both overall survival and relapse risk within a homogeneous Taiwanese AML cohort. Importantly, our findings highlight the potential benefits of model retraining to optimize prognostic accuracy by tailoring Stellae-123 to the unique characteristics of the Taiwanese patient population. Furthermore, the integration of age contributes to refine the current risk stratification. Given the remarkable extrapolation of the signature, it should be contemplated for incorporation in the risk stratification of AML patients eligible for intensive therapy.

### Supplementary Information

Supplementary Information.

## Data Availability

The datasets generated during and/or analyzed in the present study are accessible through Gene Expression Omnibus database (accession number GSE253086) or on reasonable request from the corresponding authors.
