# Supplementary material for: Stellae-123 gene expression signature improved risk stratification in Taiwanese acute myeloid leukemia patients
Source: Sci Rep. 2024 May 14;14:11064. doi: 10.1038/s41598-024-61022-5 (PMC11094146; doi:10.1038/s41598-024-61022-5)

Supplemental material

Supplemental Method

Supplemental Tables 1-8

Supplemental Figures 1-3

Supplemental method - Library preparation, RNA sequencing, and analysis pipeline

RNA was extracted from fresh BM samples of 340 patients and sent for RNA sequencing. The TruSeq Stranded mRNA Library Prep Kit (Illumina, San Diego, CA, USA) was used for library preparation, as previously described(1). Briefly, first-strand cDNA was synthesized using reverse transcriptase and random primers. After the generation of double-strand cDNA and adenylation on the 3' ends of DNA fragments, adaptors were ligated and then purified with the AMPure XP system (Beckman Coulter, Beverly, USA). Library quality was assessed using an Agilent Bioanalyzer 2100 system and a real-time polymerase chain reaction (PCR) system. Libraries were sequenced (Genomics, BioSci & Tech Co., Taiwan) on an Illumina NovaSeq 6000 platform with 150 bp paired-end reads generated by Genomics, BioSci & Tech Co., New Taipei City, Taiwan. Low quality bases and adapter sequences in the raw data were removed using Cutadapt (v 3.0)(2). Reads were then aligned to the human genome (GRCh38) and gene annotated with its corresponding GTF files (GENCODE GRCh38) using STAR version 2.4.2a with the settings –outFilterMultimapNmax 20 –outFilterType BySJout –alignSJoverhangMin 8 –quantMode GeneCounts(3). DESeq2 was used to perform differential gene expression analysis and to calculate FPKM (fragments per kilobase of transcript per million mapped reads) values for each gene, counting only reads that mapped to exonic regions(4). Genes were called as differentially expressed if they exhibited a Benjamini and Hochberg–adjusted P value (FDR) below 0.05 and a mean fold change of above 2.

Reference

1. Wang Y-H, Hou H-A, Lin C-C, Kuo Y-Y, Yao C-Y, Hsu C-L, et al. A CIBERSORTx-based immune cell scoring system could independently predict the prognosis of patients with myelodysplastic syndromes. Blood Advances. 2021;5(22):4535-48.
2. Martin M. Cutadapt removes adapter sequences from high-throughput sequencing reads. 2011. 2011;17(1):3.
3. Dobin A, Davis CA, Schlesinger F, Drenkow J, Zaleski C, Jha S, et al. STAR: ultrafast universal RNA-seq aligner. Bioinformatics. 2013;29(1):15-21.
4. Love MI, Huber W, Anders S. Moderated estimation of fold change and dispersion for RNA-seq data with DESeq2. Genome Biol. 2014;15(12):550.

Supplemental Table 1. Diagnosis of 304 patients with acute myeloid leukemia (AML) based on the International Consensus Classification

**International Consensus Classification**		
Diagnosis	Number	Percentage
AML not otherwise specified (NOS)	46	15.1
AML with in-frame bZIP *CEBPA* mutations	58	19.1
AML with inv(16)(p13.1q22) or t(16;16)(p13.1;q22)/*CBFB*::*MYH11*	17	5.6
AML with inv(3)(q21.3q26.2) or t(3;3)(q21.3;q26.2)/*GATA2*; *MECOM*(*EVI1*)	4	1.3
AML with mutated *NPM1*	64	21.1
AML with mutated *TP53*	9	3
AML with myelodysplasia-related cytogenetic abnormalities	16	5.3
AML with myelodysplasia-related gene mutations	46	15.1
AML with other *KMT2A* rearrangements	4	1.3
AML with other rare recurring translocations	3	1
AML with t(6;9)(p22.3;q34.1)/*DEK*::*NUP214*	2	0.7
AML with t(8;21)(q22;q22.1)/*RUNX1*::*RUNX1T1*	31	10.2
AML with t(9;11)(p21.3;q23.3)/*MLLT3*::*KMT2A*	4	1.3

Supplemental Table 2. Second-line Treatment of 71 patients who did not achieve complete remission after induction chemotherapy

Treatment	Number (%)	Response / outcome	Number (%) in each regimen category
High dose cytarabine (HDAC)*	10 (14.1)	PR	4 (40)
		Refractory	6 (60)
Anthracycline with cytarabine (3+7/2+5)	8 (11.3)	CR	2 (25)
		PR	3 (37.5)
		Refractory	3 (37.5)
Anthracycline + HDAC (I1~2 HDAC)†	7 (9.9)	PR	4 (57)
		Refractory	2 (29)
		Death due to comorbidity	1 (14)
Mitoxantrone + cytarabine (N3A7)††	5 (7)	PR	1 (20)
		Refractory	4 (80)
Mitoxantrone + HDAC base¶	23 (32.4)	CR	8 (35)
		PR	3 (13)
		Refractory	11 (48)
		Death due to comorbidity	1 (4)
Mitoxantrone + etoposide + cytarabine (NEC)§	6 (8.5)	CR	1 (17)
		PR	1 (17)
		Refractory	4 (66)
Fludarabine, high dose cytarabine, and granulocyte-colony stimulating factor (FLAG)	3 (4.2)	CR	1 (33)
		PR	1 (33)
		Refractory	1 (33)
Hematopoietic stem cell transplant	2 (2.8)	CR	2 (100)
Palliative chemotherapy#	5 (7)	Refractory	5 (7)
No second-line treatment	2 (2.8)	Patient loss follow up	2 (2.8)

* High dose cytarabine (HDAC): 2 g/m^2 given q12h for 4-5 days, 8 doses in total

† Idarubicin 12 mg/m^2 daily for 1 or 2 days with HDAC for 4-5 days

†† Mitoxantrone 6 mg/m^2 given for 3 days with standard dose of cytarabine (100-200 mg/m^2) for 7 days

¶ Mitoxantrone 6 mg/m^2 given for 2 or 3 days with HDAC for 4-5 days

§ Mitoxantrone 6 mg/m^2 + etoposide 80 mg/m^2 + cytarabine 1 g/m^2 given daily for 6 days

Include low-dose cytarabine at 20 mg once or twice daily for 10 consecutive days or oral etoposide

Supplemental Table 3. Mutation profile of patients in different Stellae-123 risk groups

Variable [presented as n (%)]	Total	Stellae-123 Risk Group Favorable (n=101)	Intermediate (n=101)	Adverse (n=102)	*p* value
NPM1	64 (21.1)	24 (23.8)	25 (24.8)	15 (14.7)	NS
CEBPA					
Any mutations	69 (22.7)	47 (46.5)	19 (18.8)	3 (2.9)	<0.001
Double mutations	52 (17.1)	38 (37.6)	14 (13.9)	0 (0)	<0.001
bZIP in-frame mutations	59 (19.4)	42 (41.6)	16 (15.8)	1 (1)	<0.001
FLT3-ITD	74 (24.3)	9 (8.9)	39 (38.6)	26 (25.5)	<0.001
FLT-TKD	28 (9.2)	8 (7.9)	10 (9.9)	10 (9.8)	NS
ASXL1	23 (7.6)	0 (0)	2 (2)	21 (20.6)	<0.001
BCOR	4 (1.3)	1 (1)	2 (2)	1 (1)	NS
c-CBL	3 (1)	0 (0)	1 (1)	2 (2)	NS
DNMT3A	52 (17.1)	15 (14.9)	20 (19.8)	17 (16.7)	NS
ETV6	7 (2.3)	0 (0)	4 (4)	3 (2.9)	NS
GATA2	36 (11.8)	17 (16.8)	9 (8.9)	10 (9.8)	NS
IDH1	16 (5.3)	4 (4)	6 (5.9)	6 (5.9)	NS
IDH2	34 (11.2)	10 (9.9)	9 (8.9)	15 (14.7)	NS
JAK2	1 (0.3)	0 (0)	1 (1)	1 (1)	NS
KIT	16 (5.3)	4 (4)	8 (7.9)	4 (3.9)	NS
KRAS	11 (3.6)	4 (4)	5 (5)	2 (2)	NS
MLL	18 (5.9)	2 (2)	10 (9.9)	6 (5.9)	NS
NRAS	55 (18.1)	23 (22.8)	19 (18.8)	13 (12.7)	NS
PHF6	2 (0.7)	0 (0)	1 (1)	1 (1)	NS
PTPN11	13 (4.3)	3 (3)	2 (2)	8 (7.8)	NS
Rad21	9 (3)	4 (4)	5 (5)	0 (0)	NS
RUNX1	31 (10.2)	3 (3)	8 (7.9)	20 (19.6)	<0.001
SETBP1	3 (1)	0 (0)	0 (0)	3 (2.9)	NS
SF3B1	7 (2.3)	0 (0)	5 (5)	2 (2)	NS
SMC1A	8 (2.6)	2 (2)	3 (3)	3 (2.9)	NS
SMC3	2 (0.7)	0 (0)	2 (2)	0 (0)	NS
SRSF2	8 (2.6)	0 (0)	1 (1)	7 (6.9)	0.004
STAG1	1 (0.3)	0 (0)	1 (1)	0 (0)	NS
STAG2	7 (2.3)	0 (0)	3 (3)	4 (3.9)	NS
TET2	38 (12.5)	10 (9.9)	13 (12.9)	15 (14.7)	NS
TP53	12 (3.9)	1 (1)	2 (2)	9 (8.8)	0.008
U2AF1	11 (3.6)	2 (2)	1 (1)	8 (7.8)	0.018
WT1	29 (9.5)	12 (11.9)	12 (11.9)	5 (4.9)	NS

Supplemental Table 4. Concordance indexes of the retrained Stellae-123 model (Taiwan) for predicting overall survival (OS) and relapse-free survival (RFS) in landmark analysis.

**Landmark time after diagnosis** (number of patients in each analysis)	**Concordance index**	
	RFS	OS
1 month (289)	0.6728	0.6752
2 months (280)	0.6739	0.6811
3 months (270)	0.6699	0.6790
6 months (255)	0.6597	0.6714

Supplemental Table 5. Multivariable analysis for overall survival (OS) and relapse-free survival (RFS) in the 304 AML patients, using the pretrained model (Stellae-123 BeatAML) (upper panel) and the retrained model (Stellae-123 Taiwan) (lower panel). Stellae-123 models were tested in tertiles.

**Pretrained model (Stellae-123 BeatAML)**								
	**RFS**				**OS**			
	HR	95%CI		*p*	HR	95%CI		*p*
Age*	1.026	1.017	1.034	<0.001	1.030	1.020	1.039	<0.001
ELN-2022								
Fav (vs Adv)	0.552	0.374	0.816	0.003	0.500	0.329	0.760	0.001
Int (vs Adv)	1.124	0.785	1.609	0.523	0.990	0.685	1.432	0.958
Stellae-123 (Pretrained)								
Fav (vs Adv)	0.563	0.381	0.831	0.004	0.550	0.359	0.842	0.006
Int (vs Adv)	0.684	0.486	0.964	0.030	0.813	0.574	1.151	0.243

**Retrained model (Stellae-123 Taiwan)**								
	**RFS**				**OS**			
	HR	95%CI		*p*	HR	95%CI		*p*
Age*	1.025	1.017	1.034	<0.001	1.029	1.020	1.039	<0.001
ELN-2022								
Fav (vs Adv)	0.577	0.389	0.857	0.006	0.555	0.361	0.853	0.007
Int (vs Adv)	1.074	0.763	1.513	0.681	0.986	0.689	1.411	0.937
Stellae-123 (Retrained)								
Fav (vs Adv)	0.514	0.341	0.774	0.001	0.470	0.300	0.736	0.001
Int (vs Adv)	0.558	0.398	0.781	0.001	0.599	0.420	0.854	0.005

Statistically significant if *P*<0.05.

*As continuous variable

Abbreviations: HR, hazard ratios; CI, confidence interval.

Supplemental Table 6. Evaluation of prognostic performance in different models using the Akaike Information Criterion (AIC).

Stratification model	RFS		OS	
	AIC value	ΔAIC	AIC value	ΔAIC
ELN-2022	2273	-	2003	-
+ Stellae-123 (Beat AML)	2269	-4	1998	-5
+ Stellae-123 (Beat AML) + age	2238	-35	1961	-43
+ Stellae-123 (Taiwan)	2251	-22	1982	-22
+ Stellae-123 (Taiwan) + age	2219	-54	1945	-58

Supplemental Table 7. Concordance indexes of the prognostic system that incorporates ELN-2022, age, and the retrained Stellae-123 model (ELN/Age/AI) for predicting overall survival (OS) and relapse-free survival (RFS) in landmark analysis.

**Landmark time after diagnosis** (number of patients in each analysis)	**Concordance index**	
	RFS	OS
1 month (289)	0.7297	0.7355
2 months (280)	0.7282	0.7390
3 months (270)	0.7179	0.7331
6 months (255)	0.7073	0.7257

Supplemental Table 8. Mutation profile of patients in the ELN/Age/AI risk groups

Variable [presented as n (%)]	Total	ELN/Age/AI Risk Group Favorable (n=101)	Intermediate (n=101)	Adverse (n=102)	*p* value
NPM1	64 (21.1)	17 (16.8)	23 (22.8)	24 (23.5)	NS
CEBPA					
Any mutations	69 (22.7)	49 (48.5)	13 (12.9)	7 (6.9)	<0.001
Double mutations	52 (17.1)	38 (37.6)	11 (10.9)	3 (2.9)	<0.001
bZIP in-frame mutations	59 (19.4)	45 (44.6)	11 (10.9)	3 (2.9)	<0.001
FLT3-ITD	74 (24.3)	12 (11.9)	25 (24.8)	37 (36.3)	<0.001
FLT-TKD	28 (9.2)	10 (9.9)	7 (6.9)	11 (10.8)	NS
ASXL1	23 (7.6)	4 (4)	4 (4)	15 (14.7)	0.004
BCOR	4 (1.3)	1 (1)	1 (1)	2 (2)	NS
c-CBL	3 (1)	0 (0)	1 (1)	2 (2)	NS
DNMT3A	52 (17.1)	7 (6.9)	23 (22.8)	22 (21.6)	0.004
ETV6	7 (2.3)	2 (2)	1 (1)	4 (3.9)	NS
GATA2	36 (11.8)	20 (19.8)	9 (8.9)	7 (6.9)	0.009
IDH1	16 (5.3)	2 (2)	6 (5.9)	8 (7.8)	NS
IDH2	34 (11.2)	7 (6.9)	9 (8.9)	18 (17.6)	0.036
JAK2	2 (0.7)	0 (0)	1 (1)	1 (1)	NS
KIT	16 (5.3)	10 (9.9)	4 (4)	2 (2)	0.031
KRAS	11 (3.6)	3 (3)	4 (4)	4 (3.9)	NS
MLL	18 (5.9)	0 (0)	10 (9.9)	8 (7.8)	0.007
NRAS	55 (18.1)	25 (24.8)	19 (18.8)	11 (10.8)	0.034
PHF6	2 (0.7)	0 (0)	0 (0)	2 (2)	NS
PTPN11	13 (4.3)	3 (3)	3 (3)	7 (6.9)	NS
Rad21	9 (3)	5 (5)	2 (2)	2 (2)	NS
RUNX1	31 (10.2)	1 (1)	5 (5)	25 (24.5)	<0.001
SETBP1	3 (1)	0 (0)	0 (0)	3 (2.9)	NS
SF3B1	7 (2.3)	0 (0)	0 (0)	7 (6.9)	0.001
SMC1A	8 (2.6)	2 (2)	3 (3)	3 (2.9)	NS
SMC3	2 (0.7)	2 (2)	0 (0)	0 (0)	NS
SRSF2	8 (2.6)	0 (0)	0 (0)	8 (7.8)	<0.001
STAG1	1 (0.3)	0 (0)	0 (0)	1 (1)	NS
STAG2	7 (2.3)	1 (1)	1 (1)	5 (4.9)	NS
TET2	38 (12.5)	9 (8.9)	11 (10.9)	18 (17.6)	NS
TP53	12 (3.9)	1 (1)	2 (2)	9 (8.8)	0.008
U2AF1	11 (3.6)	1 (1)	1 (1)	9 (8.8)	0.003
WT1	29 (9.5)	6 (5.9)	17 (16.8)	6 (5.9)	0.009

Supplemental Figures

Figure legend

Supplemental Figure 1. Kaplan-Meier survival curves of overall survival (A) and relapse-free survival (B) of Taiwanese patients according to European LeukemiaNet (ELN)-2022 risk groups. Fav: favorable, Int: intermediate, and Adv: adverse.

Supplemental Figure 2. Landmark analysis demonstrating relapse-free survival in the retrained Stellae-123 model (Taiwan). Landmark time was set at 1 (A), 2 (B), 3 (C), and 6 (D) months after diagnosis, respectively.

Supplemental Figure 3. Landmark analysis demonstrating overall survival and relapse-free survival in the ELN/Age/AI (Stellae-123 retrained model) system. Landmark time was set at 1 (A), 2 (B), 3 (C), and 6 (D) months after diagnosis, respectively.

Supplemental Figure 1.

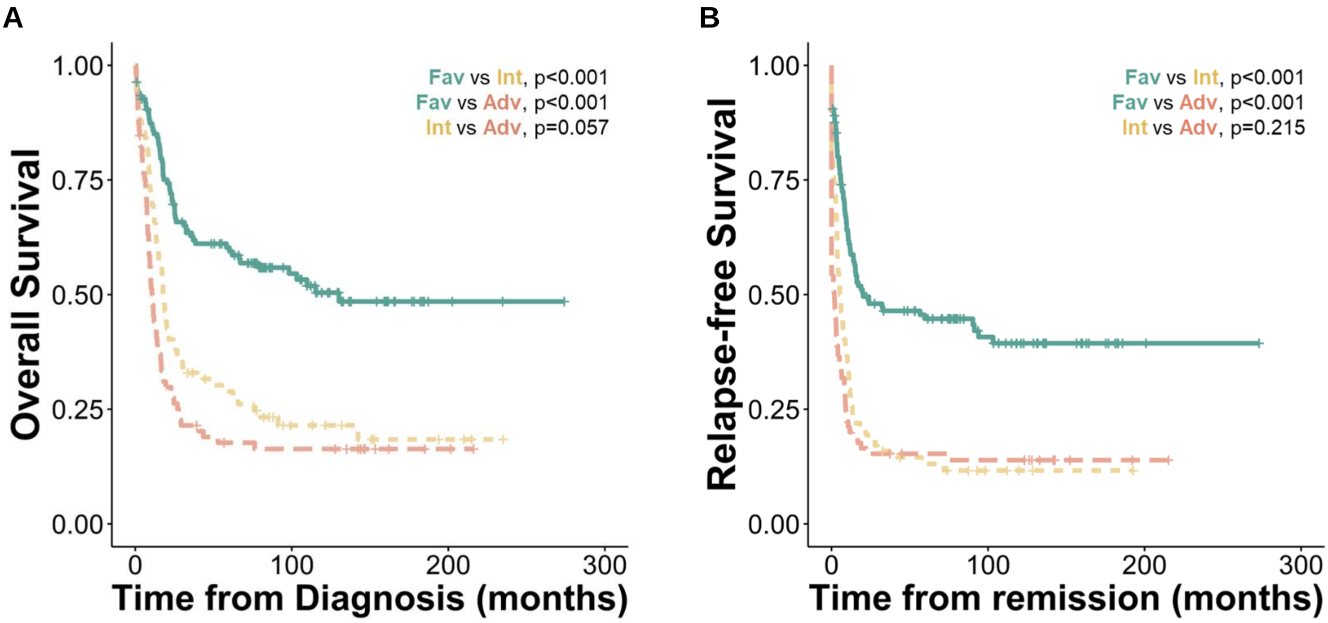

Supplemental Figure 2.

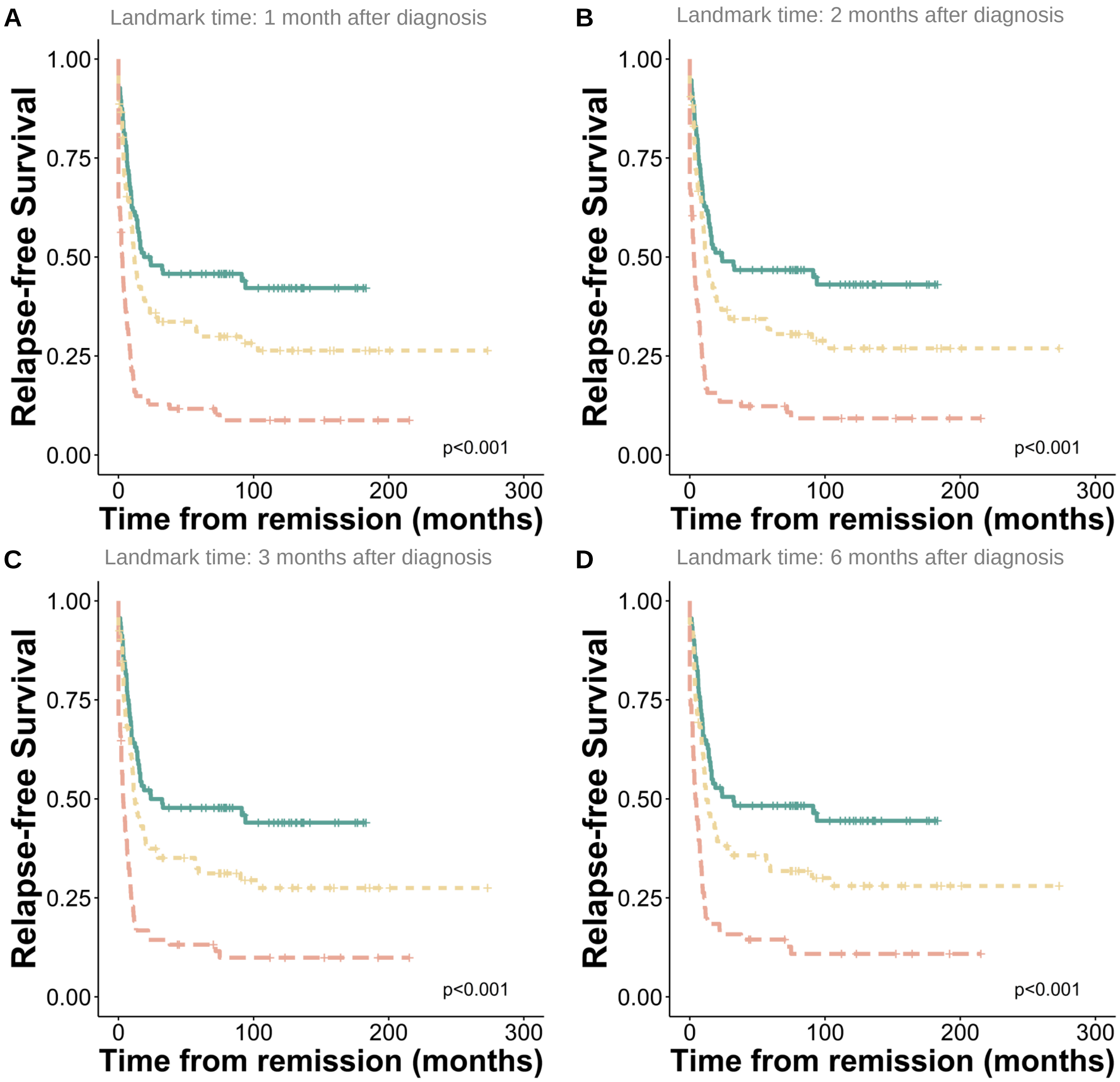

Supplemental Figure 3.

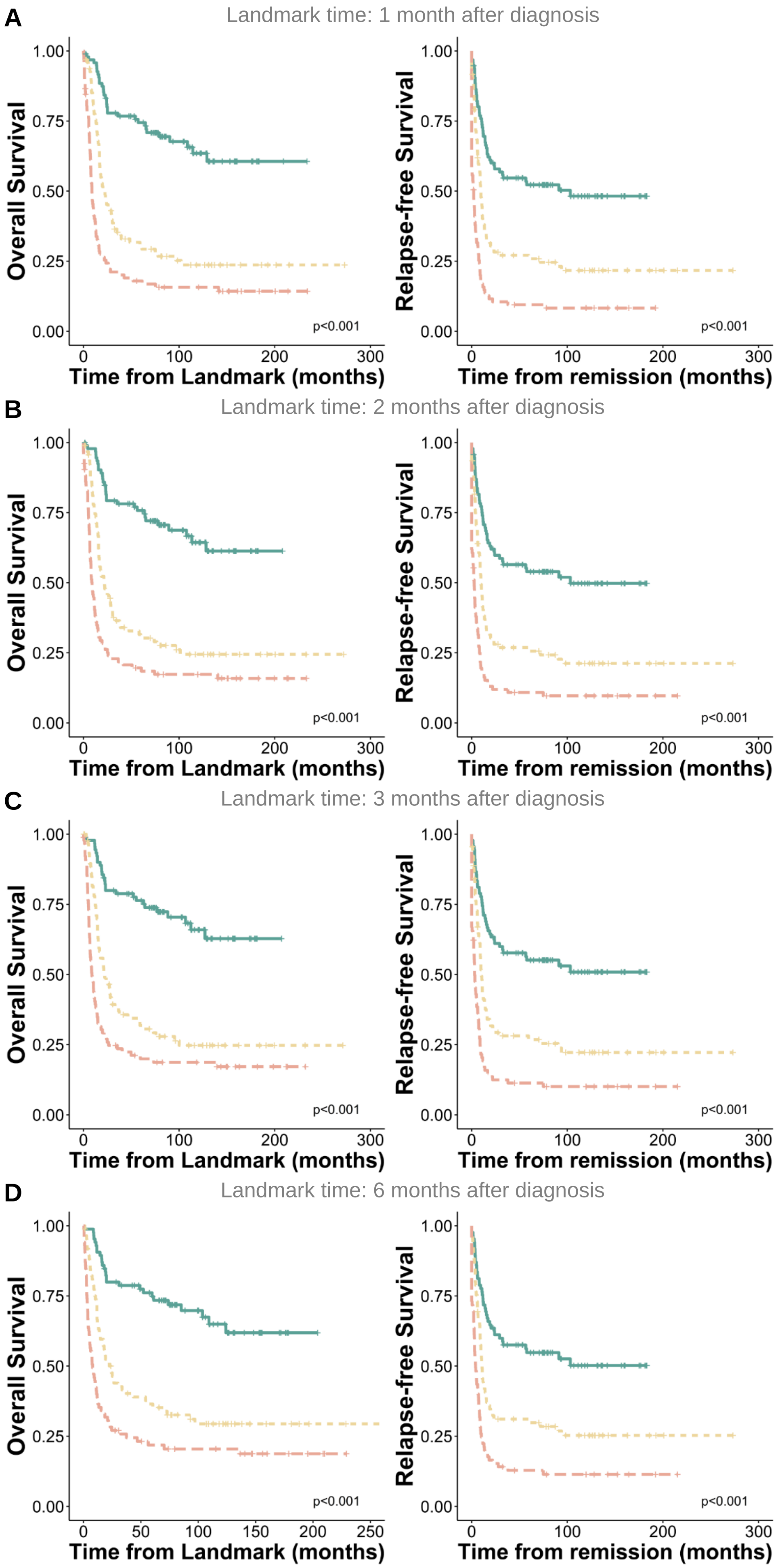

Supplement: Supplementary file 1 — Supplementary Information. [file 41598_2024_61022_MOESM1_ESM.pdf]
